# Time-window of early detection of response to concurrent chemoradiation in cervical cancer by using diffusion-weighted MR imaging: a pilot study

**DOI:** 10.1186/s13014-015-0493-6

**Published:** 2015-09-04

**Authors:** Ying Liu, Haoran Sun, Renju Bai, Zhaoxiang Ye

**Affiliations:** Department of Radiology, Tianjin Medical University Cancer Institute and Hospital, National Clinical Research Center of Cancer, Key Laboratory of Cancer Prevention and Therapy, Tianjin, China; Department of Radiology, Tianjin Medical University General Hospital, Tianjin, China

## Abstract

**Background:**

To investigate the feasibility of DWI in evaluating early therapeutic response of uterine cervical cancer to concurrent chemoradiation (CCR) and establish optimal time window for early detection of treatment response.

**Methods:**

This was a prospective study and informed consent was obtained from all patients. Thirty-three patients with uterine cervical cancer who received CCR underwent conventional MRI and DWI examinations prior to therapy (base-line) and at 3 days (postT1), 7 days (postT2), 14 days (postT3), 1 month (postT4) and 2 months (postT5) after the therapy initiated. Tumor response was determined by comparing the base-line and postT5 MRI by using RECIST criterion.

**Results:**

Percentage ADC change (γADC) of complete response (CR) group at each follow up time was greater than that of partial response (PR) group, and the differences were significant at postT3 (*p* = 0.007), postT4 (*p* = 0.001), and postT5 (*p* = 0.019). There was positive correlation between γADC at each follow-up time and percentage size reduction at postT5. The day of 14 after the therapy initiated can be considered as the optimal time for monitoring early treatment response of uterine cervical cancer to CCR, and the representative and sensitive index was γADC. With the cut-off value of 35.4 %, the sensitivity and specificity for prediction of CR group were 100 % and 73.1 %, respectively.

**Conclusions:**

It is feasible to use DWI to predict and monitor early treatment response in patients with uterine cervical cancer that undergoing CCR, and optimal time window for early detection of tumor response is the day of 14 after therapy initiated.

## Background

Uterine cervical cancer remains to be the second most prevalent gynaecological tumors worldwide [1]. Recently, cisplatin-based concurrent chemoradiation (CCR) has been established as being more effective than radiation therapy (RT) alone because chemotherapy has been shown to increase the sensitivity of tumor cells to radiation and to control both local and systemic disease manifestations [2]. However, not all tumors of the same pathological type and FIGO stage will follow the same course of disease; individual responses to CCR have been shown to vary significantly. Therefore, precise assessing tumor response before or at an early stage of CCR is important for treatment planning and for determining the prognosis.

Tumor response to therapy is conventionally assessed by changes in tumor size according to the Response Evaluation Criteria in Solid Tumors (RECIST) using computed tomography (CT) or Magnetic resonance (MR) imaging. Unfortunately, the information from post-therapy imaging after treatment completion usually comes too late to realistically impact patient management [3]. If therapy failure can be predicted effectively and as early as possible in the initial course of treatment, it will provide a window of opportunity to change the initial therapy approach upfront and clinical management can be profoundly impacted in the individual patient [3]. Given this difficulty, increasing demands are placed on imaging modalities to provide an early and reliable response marker which would have great clinical importance for uterine cervical cancer patients to cease ineffective treatment and to avoid delays in starting alternative, potentially more effective treatments.

Diffusion-weighted MR imaging (DWI) is a functional MR imaging technique that can explore the random diffusion motion of water molecules *in vivo* [4]. Previous studies in a variety of tumor types have suggested that quantitative interpretation of apparent diffusion coefficient (ADC) can be used as a biomarker for response to treatment [5–9]. To date, a few clinical studies on the usefulness of DWI as a measurement of treatment response in uterine cervical cancer have been reported [10–13]. However, systematic investigation to evaluate the optimal time window to detect early response of tumor to CCR is lacking. The present study was therefore designed to systematically analyze dynamic changes of ADC after initiation of CCR, and determine whether ADC measurements of uterine cervical cancer before and after early initiation of CCR can be used to follow treatment response, in particular, to provide a time-window of early detection of response to CCR.

## Methods

### Patient population

Our study received institutional ethics committee approval (Tianjin Medical University General Hospital) and written informed consent was obtained from all patients. Forty-five patients with biopsy-proven uterine cervical cancer, who planned to receive CCR, were prospectively recruited to this study. Inclusion criteria consisted of [a] histologically (biopsy) proven squamous cell carcinoma of uterine cervix before the first MR examination, and the time interval between biopsy and base-line MR examination did not exceed 1 month; [b] FIGO stage based on clinical examination ranges from II to IV; [c] no previous radiation or CCR treatment for uterine cervical cancer; [d] treatment consisting of radiotherapy and cisplatin-based chemotherapy; [e] no contraindications for MR examination. Exclusion criteria consisted of [a] unable to complete the full course of treatment, [b] time interval between base-line MRI and start of treatment is more than a week, [c] fail to complete the follow-up MRI examinations on time. All participants were scheduled to receive six MR examinations: before CCR (base-line), at 3 days (postT1), 7 days (postT2), 14 days (postT3), 1 month (postT4) and 2 months (postT5) after therapy initiated. 12 patients were excluded from the study because of unable to complete the full course of treatment (5 patients) or fail to complete the follow-up MRI examinations on time owing to patient incompliance (7 patients). Finally, 33 female patients (mean age 53.6 years; age range, 36–75 years) with uterine cervical cancer enrolled in this study.

### Treatment

All patients were scheduled to undergo external beam radiation therapy (EBRT) of the pelvis and intracavitary brachytherapy (ICBT). Treatment was composed of 2 days per week of brachytherapy in the form of intracavitary (60 Gy/12 fractions), and then 3 days per week of pelvic external beam radiotherapy (42 Gy/21 fractions to point B), accompanied with cisplatin chemotherapy at a dose of 40 mg/m^2^ during the intervening weekends.

### MR examination

All MR examinations were performed using a 1.5-T unit (Twin Excite, GE Healthcare, USA) with a torso phased-array body coil. Before DWI, conventional T2-weighted fast spin-echo in the sagittal and transverse planes (TR/TE, 4,000 ms/85 ms; matrix size, 320 × 224; band width, 31.25 Hz/pixel; field of view, 36 cm; number of excitations, 2; slice thickness, 6 mm; gap, 1 mm), T2-weighted fast spin-echo with fat suppression in the transverse plane (the parameters were the same as for the T2-weighted image) and T1-weighted spin-echo in the transverse plane (TR/TE, 500 ms/20 ms; matrix size, 320 × 160; band width, 31.25 Hz/pixel; field of view, 36 cm; number of excitations, 2; slice thickness, 6 mm; gap, 1 mm) were obtained.

Diffusion-weighted MR images were acquired using a non-breath-hold single-shot spin-echo echo-planar imaging (EPI) sequence and array spatial sensitivity encoding technique (ASSET) in the transverse plane (TR/TE, 4,000 ms/58.5 ms for b values of 0 and 1000 s/mm^2^; matrix size, 128 × 128; field of view, 36 cm; number of excitations, 4; slice thickness, 6 mm; gap, 1 mm; R factor, 2; phase-encoding direction, anteroposterior). The diffusion-weighting gradients were applied in all three orthogonal directions. The scanning time of DWI was 1 min and 4 s.

### MR image analysis

MR images were analyzed by two radiologists who performed tumor ADC measurements and longest tumor diameter measurement on the pre- and post-CCR images independently. The readers were blinded to each other’s results. The longest tumor diameter was measured using the transverse plane on T2-weighted images. ADC maps were calculated on a pixel-by-pixel basis by using built-in software (AW4.3 Functool; GE Healthcare). For ADC calculation, up to three slices depicting the largest tumor diameter were selected and then tumor margins were free-hand delineated on DW images. In each slice a region of interest (ROI) was delineated according to the tumor geometry. The border of the ROI was placed in the tumor periphery close to the tumor margin in order to encompass as much of the tumor as possible with exclusion of hemorrhagic or necrotic areas. If the residual tumor was tiny and covered less than three slices, the ROIs were assessed twice in the same site by each experienced radiologist. In case of invisible residual tumors on MR images after completion of therapy at T5, the ROI was drawn on the same area where tumors were identified at base-line MR images. Mean ADC value was calculated by average for each tumor.

The ADC changes (γADC) (as a percentage) for each lesion between the base-line and follow-up time points were calculated using the formula:

$$ \upgamma \mathrm{ADC}=\left(\mathrm{AD}{\mathrm{C}}_{\mathrm{F}}-\mathrm{AD}{\mathrm{C}}_{\mathrm{B}}\right)/\mathrm{AD}{\mathrm{C}}_{\mathrm{B}}\times 100, $$

where ADC_B_ represents pretreatment ADC values, and ADC_F_ represents the post-treatment ADC values after CCR initiated.

The longest tumor diameter changes (γD) (as a percentage) for each lesion between the base-line and follow-up time points were calculated using the formula:

$$ \upgamma \mathrm{D}=\left({\mathrm{D}}_{\mathrm{B}}-{\mathrm{D}}_{\mathrm{F}}\right)/{\mathrm{D}}_{\mathrm{B}}\times 100, $$

where D_B_ represents pretreatment longest tumor diameter, and D_F_ represents the post-treatment longest tumor diameter after CCR initiated.

### Treatment outcome analysis

According to Response Evaluation Criteria in Solid Tumors (RECIST 1.1), response to treatment was determined by comparing the base-line MRI and follow-up MRI at 2 months after the therapy initiated [14]. Complete response (CR) was concluded if there was no residual tumor on T2-weighted images; partial response (PR) was concluded if the longest diameter of the tumor was less than 70 % of the original size; the disease was determined to be stable (SD) if there was neither sufficient shrinkage to qualify for partial response nor sufficient increase to qualify for progressive disease; and progressive disease (PD) was concluded if there was at least a 20 % increase in the longest diameter of tumor, taking as reference the longest diameter recorded pre-treatment.

### Statistical analysis

Statistical analyses were performed using the Statistical Package for the Social Sciences (SPSS, version 13.0). Intra-class correlation (ICC) of coefficient between two readers was calculated. Multiple comparisons of continuous data were performed by Randomized blocks analysis of variance. In order to test differences between two independent groups, statistical comparisons were made using Student’s *t*-test. Pearson correlation coefficient was used in order to test independence between variables. A ROC analysis was performed to determine the optimal time window for early detection of tumor response to CCR. From the ROC analysis, Youden’s index was used in determining the optimal threshold value. The resulting threshold values were then used to calculate the sensitivity and specificity. A level of *p* value <0.05 was regarded as statistically significant.

## Results

The agreement of two readers was excellent with an ICC of 0.82 for the assessment of tumor diameter and 0.85 for ADC values.

### Patient diagnosis and outcome

**Fig. 1:**
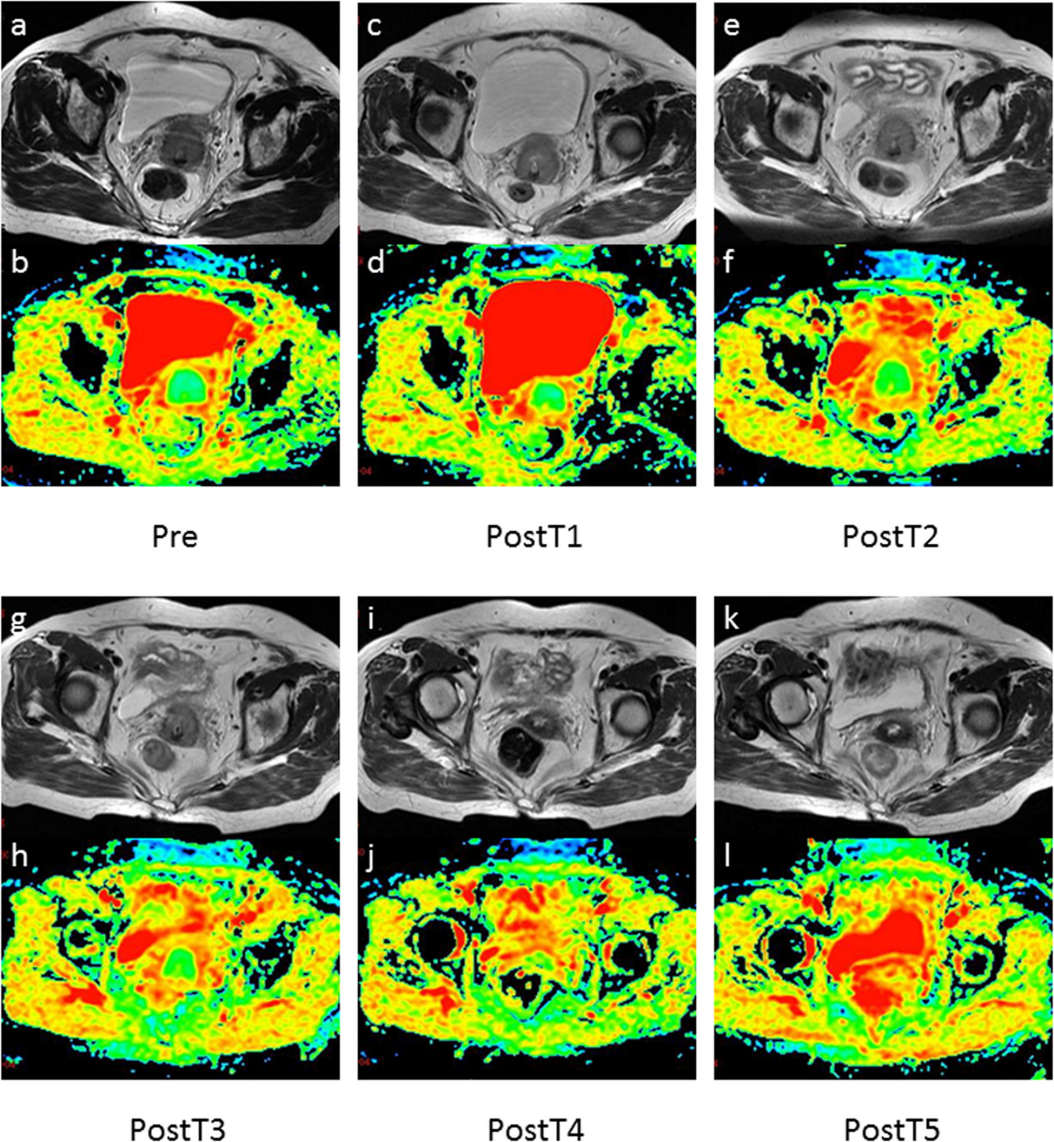
MRI and DWI images of uterine cervical cancer in group CR ((**a–b**), before CCR; (**c–d**), postT1; (**e–f**), postT2; (**g–h**), postT3; (**i–j**), postT4; (**k–l**), postT5). At base-line MRI, axial T2-weighted images (**a**) show a hyperintense tumor with well-defined margin before therapy. At postT1 (**c**) and postT2 (**e**), no significant change in size was shown. At postT3 (**g**), tumor showed a decrease in size. At postT4, tumor reduced in size markedly (**i**). At postT5, no residual tumor could be seen on T2-weighted images (**k**). The ADC values were 0.816 × 10^−3^ mm^2^/s at base-line (**b**), 0.935 × 10^−3^ mm^2^/s at postT1 (**d**), 1.090 × 10^−3^ mm^2^/s at postT2 (**f**), 1.110 × 10^−3^ mm^2^/s at postT3 (**h**),1.280 × 10^−3^ mm^2^/s at postT4 (**j**), and 1.350 × 10^−3^ mm^2^/s at postT5 (l)

33 female patients with squamous cell carcinoma of uterine cervix were enrolled in this study, including 10 cases of IIb, 1 case of IIIa and 22 cases of IIIb. Mean pretreatment diameter of tumor was 45.6 mm (range, 26.7–89.6 mm). Standard radiographic follow up of tumor response classified 7 patients as CR (Fig. 1), 26 patients as PR.

### Comparison of base-line ADC and tumor diameter between CR and PR

The base-line tumor diameter of group PR was slightly larger than group CR, but there was no significant difference between them (*t* = −0.443, *p* = 0.661). The base-line ADC value of group CR was significantly lower than that of PR (*t* = −2.991, *p* = 0.007) (Table 1).

**Table 1:** Pre-treatment ADC value and tumor diameter of CR and PR

	Pre-treatment ADC(10^−3^ mm^2^/s)	Pre-treatment tumor diameter (mm)
CR (*n* = 7)	0.810 ± 0.015	43.400 ± 7.465
PR (*n* = 26)	0.863 ± 0.088	46.223 ± 16.260

The mean base-line ADC value of all tumors was (0.852 ± 0.081) × 10^−3^ mm^2^/s, mean base-line tumor diameter was (45.624 ± 14.778) mm and mean percentage size reduction of tumor at postT5 was (65.9 ± 20.4) %. No correlations were noted between the pre-treatment tumor diameter and pre-treatment ADC value (Pearson coefficient, −0.317; *p* = 0.072). The percentage size reduction of tumor at postT5 was significantly and inversely correlated with base-line ADC value (Pearson coefficient, −0.351; *p* = 0.045); while there was no significant correlation between the percentage size reduction of tumor at T5 and base-line tumor diameter (Pearson coefficient, −0.119; *p* = 0.508).

### Comparison of pre- and post-treatment ADC and tumor diameter

For group CR, the ADC value increased gradually after therapy initiated, and pre-treatment and post-treatment ADC value varied significantly (*p* < 0.001). Statistical difference was observed between either two times (*p* < 0.001) except for postT1 and postT2 (*P* = 0.065).

For group PR, the ADC value increased gradually after therapy initiated, and pre-treatment and post-treatment ADC value varied significantly (*p* < 0.001). Statistical difference was observed between either two times (*p* < 0.001).

The longest tumor diameter decreased gradually for both group CR and PR, and pre-treatment and post-treatment tumor diameter varied significantly (*p* < 0.001; *p* < 0.001). Tumor diameter shrinkage that achieved statistical difference at the earliest follow up time was T4 compared with pre-treatment tumor diameter for both CR and PR group.

### Comparison of ADC and γADC during treatment between CR and PR

**Fig. 2:**
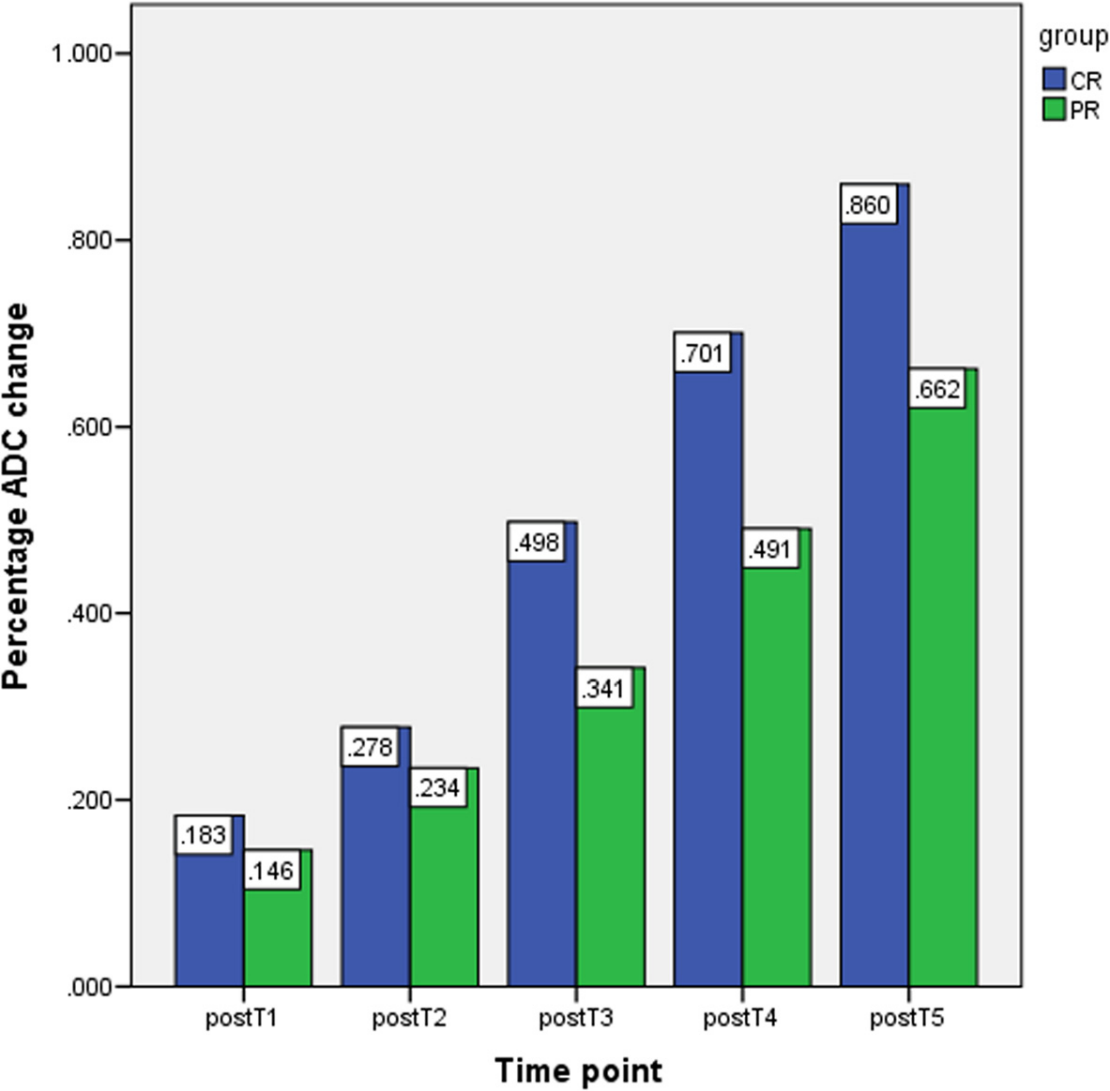
Graph shows that comparison of percentage ADC change (γADC) between CR and PR at each time point. Mean value was labeled at the top of the bar. There was no significant difference in γADC at postT1 (*P* = 0.286) or postT2 (*P* = 0.357), while γADC in CR group was significantly greater than that of PR at postT3 (*P* = 0.007), postT4 (*P* = 0.001), and postT5 (*P* = 0.019)

The ADC value for both group CR and PR increased to different extents after the initiation of CCR. For group CR, ADC values increased steadily from postT1 to postT2, and then increased sharply; while, gradual increase was found from postT1 to postT5 for group PR. There was no significant difference of ADC value between group CR and PR at either follow up time during therapy (Table 2). γADC of group CR at all follow up time was greater than that of group PR, and the differences were significant at postT3 (*P* = 0.007), postT4 (*P* =0.001), and postT5 (*p* = 0.019) (Fig. 2).

**Table 2:** Comparison of ADC value during treatment between CR and PR

	CR(10^−3^ mm^2^/s)	PR(10^−3^ mm^2^/s)	*t*	*P*
PostT1	0.958 ± 0.073	0.987 ± 0.096	−0.719	0.477
PostT2	1.036 ± 0.118	1.062 ± 0.110	−0.552	0.585
PostT3	1.213 ± 0.981	1.154 ± 0.131	1.094	0.282
PostT4	1.379 ± 0.132	1.283 ± 0.141	1.621	0.115
PostT5	1.507 ± 0.132	1.425 ± 0.140	1.389	0.175

At postT1, postT2, postT3, postT4, and postT5 there was linear positive correlation between γADC and the percentage size reduction of tumor at postT5 (*p* = 0.014, *p* = 0.026, *p* < 0.001, *p* = 0.001, *p* = 0.012). No significant correlation was found between ADC value at either follow up time and the percentage size reduction of tumor at postT5 (*p* = 0.875, *p* = 0.952, *p* = 0.187, *p* = 0.211, *p* = 0.240).

**Fig. 3:**
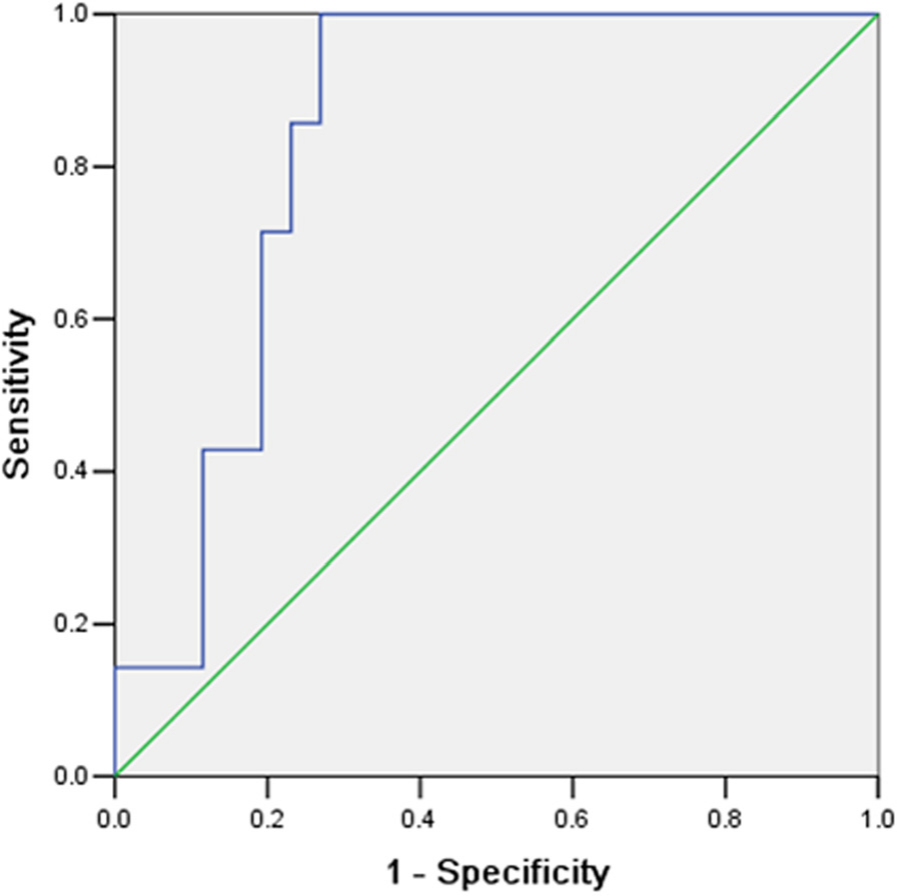
Graph shows that the ROC curve for predicting the early treatment response by using γADC. With the cut-off value of 35.4 %, the sensitivity and specificity for predicting tumor response group of CR were 100 % and 73.1 % respectively

As the difference of γADC between CR and PR group achieved significance as early as 14 days after CCR initiated, and meanwhile γADC of cervical cancer had a significant linear correlation with tumor response at that time; the day of 14 after the therapy initiated can be used as the optimal time window for early detection of tumor response to CCR, and the representative and sensitive index for evaluation therapeutic efficacy was γADC. A cut-off value was calculated using γADC by ROC analysis (Fig. 3). With the percentage ADC change of 35.4 %, the sensitivity and specificity for predicting a MRI complete response were 100 % and 73.1 % respectively (95 % confidence interval, 0.708–0.974) and the area under the ROC curve was 0.841.

### Comparison of tumor diameter and γD during treatment between CR and PR

The tumor diameter decreased gradually for both group CR and PR, but there were no significant differences of tumor diameter or γD between them at either follow up time during therapy (Tables 3 and 4).

**Table 3:** Comparison of tumour diameter during treatment between CR and PR

	CR(mm)	PR(mm)	*t*	*p*
PostT1	42.200 ± 6.590	45.385 ± 16.037	−0.509	0.614
PostT2	41.186 ± 6.228	42.638 ± 16.812	−0.222	0.826
PostT3	40.229 ± 6.187	39.246 ± 17.634	0.144	0.887
PostT4	23.957 ± 9.907	29.408 ± 17.094	−0.802	0.429

**Table 4:** Comparison of γD during treatment between CR and PR

	CR (%)	PR (%)	*t*	*p*
PostT1	2.5 ± 0.3	1.7 ± 0.2	0.741	0.464
PostT2	4.7 ± 0.6	8.6 ± 0.7	−1.305	0.202
PostT3	6.7 ± 0.8	16.8 ± 1.3	−1.962	0.059
PostT4	46.1 ± 16.6	38.2 ± 16.6	1.107	0.277

## Discussion

Conventional imaging procedures such as CT and conventional MRI evaluate the tumor response by measuring changes in tumor size caused by therapy, and it was generally accepted that a decrease in tumor size correlated with treatment effect. However, changes in morphological parameters such as volume and diameter of tumor have been used with limited success, since lesions do not substantially decrease in size in short-time after CCR and these imaging techniques may be limited in providing clinically satisfactory information about the extent of tumor necrosis, which is the main indicator of tumor cell death. Hence, there is need for new imaging modalities which can be used to satisfy the increasing demands of pretreatment prediction and early post-treatment monitoring and particularly, establish the optimal time window for early detection of tumor response to CCR [15].

DWI is a functional MR technique that has become a constant additive to morphologic imaging and its role has been already studied extensively [16]. It can be performed on most modern MRI machines without any additional new equipment or intravenous contrast agents [17]. It has become increasingly important in the assessment of tumors and evaluation of response during follow-up with various treatment modalities and it has been recommended as a cancer imaging biomarker in the clinical trials by the national cancer institute (NCI) of USA [18]. By exploiting information about changes in proton mobility caused by the alteration of tissue cellularity and the integrity of the cellular membrane, tortuosity of extracellular space, and viscosity of fluids due to pathologic processes, DWI provides a tissue contrast that is different from that made with conventional T1-weighted (T1WI) and T2-weighted images (T2WI). It can be used to characterize highly cellular and acellular regions of tumors, distinguish cystic from solid regions, as well as the change in cellularity within the tumor over time [19–21].

Several studies have confirmed that cellular tumors with low pre-treatment ADC values show a better response to various therapies than those with high pre-treatment ADC values [22, 23]. In this study, we found that the pre-treatment ADC value of group CR was significantly lower than that of group PR, in accordance with results mentioned above; moreover, pretreatment ADC value inversely correlated with the response to treatment. One possible explanation is that necrotic tumors, which are characterized by a breakdown of cellular membrane, thereby allowing free diffusion and an increase of diffusing molecules, resulting in higher ADC values, are frequently hypoxic, acidotic, and poorly perfused, leading to diminished sensitivity to chemotherapy and radiation therapy [17]. Furthermore, the distribution of chemotherapeutic agents in necrotic tumors may be less efficient because of insufficient vascularity. Therefore, it may be hypothesized that patients with necrotic areas in their tumors, and thus high pretreatment ADC values, would have a worse treatment outcome. These results demonstrate the feasibility of using DWI for pre-treatment predicting responders from non-responders in patients with uterine cervical cancer that undergoing CCR. Meanwhile, we also found that a few tumors did not respond favorably to CCR despite having lower pretreatment ADC values. The hypothesis to explain this is that, necrosis within a tumor may not always be associated with a high ADC. In theory, coagulative necrosis without tumor cell liquefaction may not increase the ADC [13]. It is therefore not adequate to use only pretreatment ADC value for response prediction since it may bring about bias. It would be preferable to have an early assessment during the course of treatment which offered a window of opportunity to optimize or alter the treatment plan in those patients who are not undergoing a satisfactory response.

Effective anticancer treatment results in tumor lysis, loss of cell membrane integrity, an increased extracellular space with a subsequent reduction in tumor cell density, which facilitates water molecule diffusion [24]. Decreases in tumor cellularity will ultimately lead to reduction in tumor size, and this reduction in tumor size can be expected after 2–3 cycles of systemic treatment, which usually is between 6 and 12 weeks after start of treatment [17]. In the present study, we observed an increase in tumor ADC value for both group CR and PR after treatment, a finding consistent with other studies [12, 25–27]. For both group CR and PR, significant alterations from the baseline value were noted for ADC value at 3 days after therapy, while changes in the measurement of the longest tumor diameter only achieved a borderline significance compared to baseline at 1 month after therapy. These data confirm that changes in ADC values precede changes in tumor size, since early after start of treatment changes in cellularity and necrosis may already occur. Thus it seems plausible that DWI had a potential ability to provide an early marker for treatment efficacy regarding microstructure changes, which may precede significant conventional morphologic alterations.

Tumor heterogeneity is seen, not just from patient to patient, but within the same primary tumor mass. It would be advantageous to accurately predict a tumor’s behavior and the time window for early detection of tumor response after the start of treatment is a key issue. Our study found that the γADC of CR was significantly higher than PR at postT3, postT4, and postT5; linear positive correlations were found between γADC and the percentage size reduction of tumor after 2 months of therapy at the time of postT1, postT2, postT3, postT4, and post T5. These results suggested that elevated ADC values may reflect the early changes within the tumor after CCR. Significantly increased ADC value after CCR may indicate chemosensitivity, however, a minute change in tumor ADC values might indicate a less satisfactory outcome or even a therapeutically unresponsive tumor, which was in line with previous results [26]. By setting up multiple time points to explore the serial change of ADC within the tumor, we found that the optimal time window for early detection of tumor response is the day of 14 after the therapy initiated, which confirmed the previous findings [26, 28, 29]; meanwhile, we found that γADC could be served as a representative and sensitive index for response assessment. With the cut-off value of 35.4 %, the sensitivity and specificity for determining the treatment response were 100 % and 73.1 % respectively. This would provide an opportunity to adjust individual treatment regimens more rapidly, and allow patients to receive the most appropriate treatment for their specific case, sparing them the unnecessary morbidity, expense, or delays in the initiation of effective treatment.

Our study has several limitations. First, the patient population is relatively small, further studies with a large number of patients are needed to confirm our preliminary results. Furthermore, we could not evaluate the exact pathological mechanism of ADC incensement during CCR. Third, tumor response is only based on tumor measurement on T2 weighted MR images at 2 months of therapy. The follow up time is relatively short, and cytology is not considered as part of the response evaluation at this time point.

## Conclusions

On the basis of our preliminary study, we conclude that it is feasible to use DWI as a non-invasive, radiation-free method to predict and monitor the early treatment response in patients with uterine cervical cancer to CCR. The day of 14 after CCR initiated can be used as the optimal time window and γADC as a reliable biomarker for early detection of tumor response, before it is clinically obvious using standard imaging modality. Early prediction of high risk to fail ongoing conventional therapy at this early time point can provide an opportunity for clinicians to change the initial therapy to more aggressive treatment regimens such as radiation dose intensification in time.

### Consent to publish

We have obtained consent to publish from the participant to report individual patient image data.

## Abbreviations

RT: Radiation therapy
FIGO: Federation of gynecology and obstetrics
CCR: Concurrent chemoradiation
RECIST: Response evaluation criteria in solid tumors
DWI: Diffusion-weighted MR imaging
ADC: Apparent diffusion coefficient
EBRT: External beam radiation therapy
ICBT: Intracavitary brachytherapy
